# Plasmonic Sensing Characteristics of Gold Nanorods with Large Aspect Ratios

**DOI:** 10.3390/s18103458

**Published:** 2018-10-15

**Authors:** Chao Zhuang, Yifan Xu, Ningsheng Xu, Jinxiu Wen, Huanjun Chen, Shaozhi Deng

**Affiliations:** State Key Laboratory of Optoelectronic Materials and Technologies, Guangdong Province Key Laboratory of Display Material and Technology, Sun Yat-sen University, Guangzhou 510275, China; zhuangch3@mail2.sysu.edu.cn (C.Z.); xuyf35@mail2.sysu.edu.cn (Y.X.); stsxns@mail.sysu.edu.cn (N.X.); jinxiuwen@foxmail.com (J.W.)

**Keywords:** large aspect ratios, gold nanorods, refractive index sensitivities, SERS, plasmonic sensing

## Abstract

Plasmonic gold nanorods play important roles in nowadays state-of-the-art plasmonic sensing techniques. Most of the previous studies and applications focused on gold nanorods with relatively small aspect ratios, where the plasmon wavelengths are smaller than 900 nm. Gold nanorods with large aspect ratios are predicted to exhibit high refractive-index sensitivity (Langmir 2008, 24, 5233–5237), which therefore should be promising for the development of high-performance plasmonic chemical- and bio-sensors. In this study, we developed gold nanorods with aspect ratios over 7.9, which exhibit plasmon resonances around 1064 nm. The refractive index (RI) sensitivity of these nanorods have been evaluated by varying their dielectric environment, whereby a sensitivity as high as 473 nm/RIU (refractive index unit) can be obtained. Furthermore, we have demonstrated the large-aspect-ratio nanorods as efficient substrate for surface enhanced Raman spectroscopy (SERS), where an enhancement factor (EF) as high as 9.47 × 10^8^ was measured using 4-methylbenzenethiol (4-MBT) as probe molecule. Finally, a type of flexible SERS substrate is developed by conjugating the gold nanorods with the polystyrene (PS) polymer. The results obtained in our study can benefit the development of plasmonic sensing techniques utilized in the near-infrared spectral region.

## 1. Introduction

Noble metal nanostructures exhibit strong localized surface plasmon resonances (LSPR), the electromagnetic modes associated with the collective oscillations of the free electrons confined to the nanoscale [1], which have gained tremendous attention in the past decades. Among the various architectures, gold nanorods elongated along their longitudinal directions have received particular interests due to that the operation wavelengths of the longitudinal LSPR, i.e., plasmon mode associated with the electron oscillations along the length axis, can be synthetically tuned across the visible to the near-infrared region by simply changing their aspect ratios, i.e., the ratio between their lengths and diameters [2]. In such a manner, the localized electromagnetic field enhancements associated with the plasmon resonances can be tailored to match the excitation wavelengths by adjusting the nanorod geometries. These unique merits have made the gold nanorods superior in various applications, especially the plasmonic sensing that is based on the dependence of the plasmon wavelengths on the surrounding refractive index, and the SERS where the plasmon-induced near-field enhancement plays an important role [3]. However, up to now, the studies and applications of the LSPR sensing and SERS mostly focused on gold nanorods with relatively low aspect ratios, which exhibit LSPR wavelengths below 900 nm [4,5,6]. From the LSPR sensing point of view, nanorod with large aspect ratios, which therefore exhibit longer plasmon resonance wavelengths, were shown to exhibit higher RI sensitivity [7,8]. For the SERS applications, the enhancements can be maximized when the excitation and Raman scattering light fields are in resonance with the LSPR wavelengths. Therefore, nanorods with longer LSPR wavelengths can enable the use of near-infrared lasers for collecting the SERS signals, which can benefit the in vivo applications. On the other hand, in the long wavelength region far away from the interband transitions of gold, the plasmon damping is very small. Thus, gold nanorods with large aspect ratios can exhibit huge electric field enhancements under resonant excitation than their counterparts with small aspect ratios, which can greatly benefit both of the LSPR sensing and SERS applications.

Recently, Ye et al. [9] had developed a seed-mediated growth method using binary surfactant, which can simultaneously improve the dimensional tunability and monodispersity of AuNRs. Using this method, gold nanorods with large aspect ratios can be readily obtained with high yield. In our current study, we further explored the LSRP sensing as well as SERS performances of these large-aspect-ratio gold nanorods. By dispersing the nanorods into aqueous solution with different refractive indices, we measured and calculated their RI sensitivity, which showed great improvements in comparison to the nanorods with small aspect ratios. In addition, the nanorods were uniformly deposited onto glass substrate to act as SERS substrate. When labelled with the 4-methylbenzenethiol (4-MBT) and polyaniline emeraldine salt form (PANI-ES) as probe molecules respectively, the SERS performances of the large-aspect-ratio gold nanorods were further evaluated. We finally showed that the gold nanorods can be furthermore integrated with the polystyrene (PS) substrates to form a flexible SERS substrate, which could be conformed to curved surface. Our results have unveiled the gold nanorods with large aspect ratios as excellent platforms in plasmonic sensing applications.

## 2. Materials and Methods

### 2.1. Chemicals

HAuCl_4_·3H_2_O (Au > 99.9%) was purchased from Aladdin (Shanghai, China). NaBH_4_ (>96%), L-ascorbic acid (AA, >99%), cetyltrimethyl ammonium bromide (CTAB) (>99%), AgNO_3_ (>99%), polyaniline emeraldine base (PANI-EB, 100 kDa), (3-Mercaptopropyl) trimethoxysilane (MPTMS, 95%), 4-methylbenzenethiol (4-MBT, 98%), N,N-dimethylacetamide (DMA, 99.8%) were purchased from Sigma-Aldrich (Milwaukee, WI, USA). Sodium oleate (NaOL) (>97%) was purchased form TCI (Shanghai, China). The HCl (37%), NaOH, and glycerol are of analytical grade. All chemicals were used without further purification. Deionized water (18.2 MΩ cm^−1^) was used during the growth of the nanorods and preparation of the samples.

### 2.2. Synthesis of the Gold Nanorods

The gold nanorods were grown using the seed-mediated method with binary surfactant (Figure S1) [9]. The seed solution was prepared by the addition of the HAuCl_4_ (5 mL, 5 mmol·L^−1^) into the CTAB (5 mL, 0.2 mol·L^−1^) in a 25 mL glass vial, followed by adding 0.6 mL of the freshly prepared NaBH_4_ (0.01 M) under vigorous stirring. The stirring was stopped after 2 min until the color of the solution changed from yellow to brownish yellow. The seed solution was aged at room temperature for 30 min before use. To prepare the growth solution, 9 g CTAB and 1.234 g NaOL were mixed in a 500 mL flask containing 250 mL 50-°C DI water, which was kept undisturbed for 15 min after the addition of the AgNO_3_ (36 mL, 4 mM). Subsequently, the HAuCl_4_ (250 mL, 1 mM) was added and stirred for 90 min, whereby the color of the mixture changed from yellow to transparent. Afterwards, the HCl solution (3.5 mL, 37 wt % in water, 12.1 M) was added to adjust the pH. The mixture was then stirred for another 15 min. The AA solution (1.25 mL, 0.064 M) was added, followed by vigorous stirring for 30 s. Finally, 0.2-mL seed solution was injected and stirred for 30 s. The mixture was then left undisturbed for 12 h for the growth of the gold nanorods. The binary surfactant method is schematically shown step-by-step in Figure S1 in the Supplementary Materials.

### 2.3. Refractive Index Sensitivity Measurements

The refractive index sensitivity measurements was similar to the previous studies [7]. Briefly, 1-mL gold nanorod solution were dispersed in water–glycerol solutions with varying glycerol volume ratios. As the volume percentage of the glycerol was varied from 0% to 90% at a step of 10%, the extinction spectra of the gold nanorods were measured sequentially and the plasmon wavelength shifts was plotted as a function of the refractive index of the water–glycerol solutions, afterwards the refractive index sensitivity was determined from linear fitting. The figure of merit is the index sensitivity divided by the full width at half-maximum (FWHM) of the extinction peak taken from aqueous dispersions of the gold nanorods.

### 2.4. Preparation of SERS Substrate on Glass

The SERS substrate on glass was prepared according to the method reported in Ref. [10]. Specifically, 5-mL of the as-grown gold nanorod solutions were centrifuged at 9500 rpm for 10 min. The precipitates were redispersed into water of identical volume, centrifuged again at 9500 rpm for another 10 min, and finally redispersed into water (5 mL). The glass substrate was first immersed into aqua regia for 1 h for the hydroxylation. After rinsing with deionized water, the glass substrate was then immersed into a 10 vol % (3-mercaptopropyl) trimethoxysilane (MPTMS) ethanol solution for 3 h to fuctionalize its surface with thiol (–SH) groups, followed by rinsed with ethanol and deionized water. The HCl was then added into the nanorod aqueous solution to adjust the pH, and subsequently the functionalized glass substrate was immersed into the nanorod solution and kept undisturbed for 6 h. Thereafter the substrate was rinsed by deionized water and blown dry with nitrogen.

### 2.5. Preparation of PANI-ES

The dispersion of PANI-ES in water/DMA was prepared according to Ref. [11]. To that end, commercially available PANI-EB with average molecular weight of 100 kDa was used to prepare the final dispersion of the PANI-ES. Firstly, 100 mg of the PANI-EB was slowly added into 5 mL of DMA under stirring. After the solution was stirred for 12 h, the solution was filtered to remove the insoluble particles. Subsequently 2 mL of the PANI-EB solution in DMA was added to 18 mL of DI water to form a dark blue solution with concentration of 0.003 M. Finally, the pH of the PANI-EB in water/DMA was carefully adjusted to 2.5 by adding the HCl (1.0 M), giving rise to a solution of dark green color, a typical characteristic of the PANI-ES solution. PANI-ES in water/DMA with concentration of 10^−3^, 10^−4^, 10^−5^, 10^−6^, and 10^−7^ M were then prepared. 100 μL of each solution was added to 20 μL of HCl (1 M), followed by the addition of 880 μL DI water. As a result, 10^−4^, 10^−5^, 10^−6^, 10^−7^, and 10^−8^ M PANI-ES diluted solution with varied pH can be obtained. To functionalize the SERS substrate on glass with the PANI-ES, 5 droplets (20 μL) of the PANI-ES solution with varying concentrations were consecutively dropped at five different positions on the SERS substrate, which were then dried in air. For comparison, the same PANI-ES solution was prepared on an empty glass substrate.

### 2.6. Functionalization of SERS Glass Substrate with 4-MBT

The SERS substrate on glass was immersed into an ethanol solution of 4-MBT (1 M) and left undisturbed for 8 h, followed by rinsing with copious amount of ethanol and DI water to remove the excessive 4-MBT molecules [12]. Finally, the substrate was blown dry by nitrogen before the Raman measurements.

### 2.7. Preparation of Flexible SERS Substrate

By spin-coating PS toluene solution onto the SERS substrate on glass, a PS film adhered with gold nanorods can be separated from the glass substrate after heating at 100 °C for 1 h [13,14]. For the Raman measurement, the PANI-ES solution was dropped onto to the PS film with gold nanorods, where the PS film was stuck to the quartz substrate with the nanorods facing with the PANI-ES molecules. The PANI-ES solution was dried naturally in ambient condition.

### 2.8. Structural and Optical Characterization

Transmission electron microscopy (TEM) images were taken on a FEI Tecnai3 G2 60–300 microscope operating at 300 kV. Topography of the SERS substrate on glass was conducted using a commercial atomic force microscope (NTEGRA Spectra). Extinction spectra were measured by HITACHI U-4100 UV-visible/NIR spectrophotometer. The Raman spectra were collected by a Renishaw in Via Reflex micro-Raman spectroscopy system using a 1064-nm laser as excitation source. The laser beam was focused onto the samples through a 50× objective (NA = 0.8), and the Raman signals were collected through the same objective in a back-scattering geometry. The laser power was kept at 1.37 mW. All spectra were obtained with an acquisition time of 60 s and integrated only 1 times.

## 3. Results and Discussion

**Figure 1:**
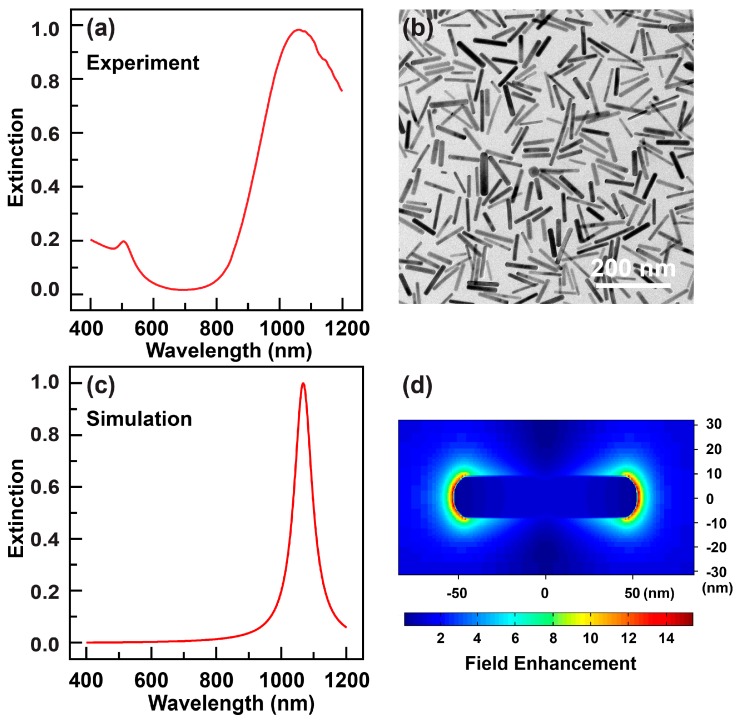
Characterizations of the gold nanorods of large aspect ratios. (**a**) Experimental extinction spectrum of the gold nanorod aqueous solution. (**b**) TEM image of a typical gold nanorod sample. (**c**) Calculated extinction spectrum of an individual gold nanorod. The geometrical parameters of the nanorod are set according to those measured from the image shown in Figure 1b. (**d**) Calculated near-field electromagnetic distributions around the individual gold nanorod. The electric fields are monitored at the extinction maximum shown in Figure 1c, and on the plane across the longitudinal axis of the nanorod.

The gold nanorods fabricated by binary surfactant seed-mediated growth method were dispersed in aqueous solution of CTAB and NaOL. The extinction spectrum of the nanorods solution was shown in Figure 1a, which indicates that the transverse plasmon mode is located at 506 nm while the longitudinal plasmon mode is centered at 1060 nm in the near-infrared region. The intensity of the longitudinal mode overwhelms that of the transverse one, suggesting that the nanorods are of very high-yield. This can be further corroborated by the TEM image of the nanorods sample (Figure 1b). From the TEM characterizations one can easily determine the length and diameter of the nanorods are 87.3 ± 14.3 nm and 11.9 ± 2.1 nm, respectively. As a result, the average aspect ratio of the nanorod is 7.9. According to the geometrical parameters provided by the TEM characterizations, we then perform numerical simulations on the electromagnetic responses of the large-aspect-ratio gold nanorod using the finite-difference time-domain (FDTD) method. As shown in Figure 1c, the longitudinal plasmon mode is well-reproduced by the simulations where the incidence polarization is parallel to the long axis of the nanorod. In addition, the simulation also reveals the strong electric field intensities distributing at the two apexes of the nanorod. Such localized electric fields can lead to distinct plasmon wavelength shifts upon change of the refractive index of the surrounding environment, as well as enhanced Raman scattering phenomena [2].

**Figure 2:**
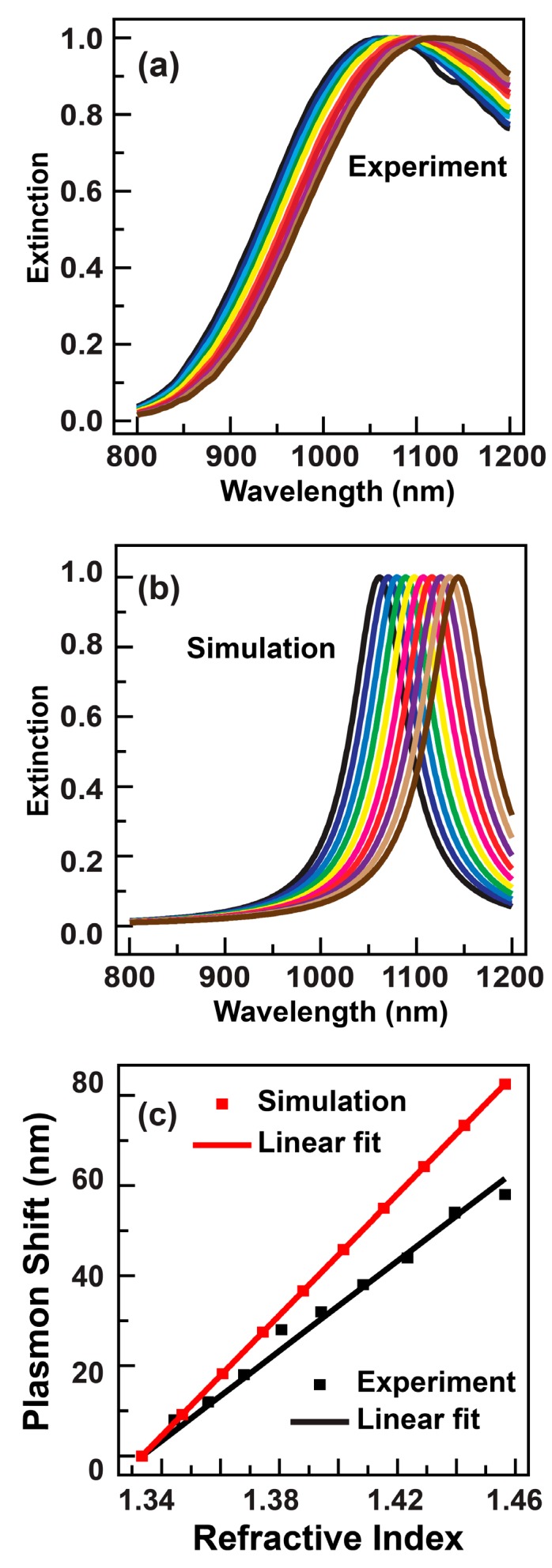
Refractive index sensitivity of the gold nanorods with large aspect ratios. (**a**) Extinction spectra of the gold nanorods stabilized in glycerol–water mixture with varied glycerol volume ratios. (**b**) Simulated extinction spectra of the gold nanorod immersed in media with varied refractive indexes. (**c**) Dependence of the longitudinal plasmon shift on the refractive index of the liquid mixture for the gold nanorods. Black and red symbols are experimental and simulated results, respectively. The lines are linear fits.

The plasmonic sensing performance was evaluated by measuring and comparing the extinction spectra of the gold nanorods stabilized in glycerol–water mixtures with varying glycerol volume ratios. The RI sensitivity can be manifested from the shift of the longitudinal plasmon mode, which is defined as the difference between the plasmon wavelengths of the gold nanorods dispersed into the liquid mixture and DI water, respectively [2]. As shown in Figure 2a, when the volume ratio of the glycerol was increased, the longitudinal plasmon peaks of the gold nanorods exhibited a redshift behavior. The longitudinal plasmon wavelengths corresponding to different mixtures can thereafter be extracted from the extinction spectra, whereby the RI sensitivity can be calculated by inspecting the dependence of the plasmon shift on the RI of the mixtures. The RI of the liquid mixture can be calculated as [15]:

(1) $$\;\frac{n_{12}^{2}-1}{n_{12}^{2}+2}=\frac{\left(n_{1}^{2}-1\right)\varphi_{1}}{n_{1}^{2}+2}+\frac{\left(n_{2}^{2}-1\right)\varphi_{2}}{n_{2}^{2}+2}\;$$

where *n*_12_ is the RI of the liquid mixture, *n*_1_ and *n*_2_ are respectively the indexes of water (1.3334) and glycerol (1.4746). The *φ*_1_ and *φ*_2_ are the volume ratios of the two components. In our study, to avoid the deviation in the glycerol volume due to the high viscosity of the glycerol, we instead calculated the RI of the mixture according to the respective masses of the two solutions [15]:

(2) $$\;\frac{n_{12}^{2}-1}{n_{12}^{2}+2}=\frac{m_{1}}{\rho_{1}\times V}\frac{n_{1}^{2}-1}{n_{1}^{2}+2}+\frac{m_{2}}{\rho_{2}\times V}\frac{n_{2}^{2}-1}{n_{2}^{2}+2}\;$$

where *V = m_1_/ρ_1_ + m_2_/ρ_2_* is the total volume of the mixture, *m*_1_ and *m*_2_ are the mass of water and glycerol, *ρ*_1_ and *ρ*_2_ are the density of water (1.000) and glycerol (1.2613), respectively. The calculated refractive index of the liquid mixture as a function of the mass of glycerol can be fitted well by a line (Supplementary Materials, Figure S2).

The plot of the plasmon shift against the RI of the mixture is shown in Figure 2c, which exhibits an evident linear behavior. The RI sensitivity can therefore be determined as 473.5 nm/RIU from the slope of the linear fit. Such a sensitivity is higher than the nanorods with shorter resonance wavelength measured before [7]. The figure of merit can thereafter be calculated to be 1.8. Such a value is comparable to those of the nanorods with small aspect ratios reported previously [7,8]. The experimental RI sensing characteristics agree well with the numerical simulations. As shown in Figure 2b, the calculated plasmon peaks of the gold nanorods shift toward the red direction along with increase of the RI of the surrounding medium. In addition, the calculated plasmon shifts exhibit a linear dependence on the RI as well (Figure 2c). However, the calculated index sensitivity (670 nm/RIU) is larger than that obtained from the experiment. Such a discrepancy is believed to be caused by the surfactant capping layer on the nanorods. The presence of the capping layer around the gold nanorods can on one hand reduce the effective RI experience by the nanorods, and on the other hand shield the localized electromagnetic fields induced by the plasmon resonance. Both of these two effects can deteriorate the plasmon shift when the mixture composition is varied, giving rise to a smaller index sensitivity measured from the experiments.

**Figure 3:**
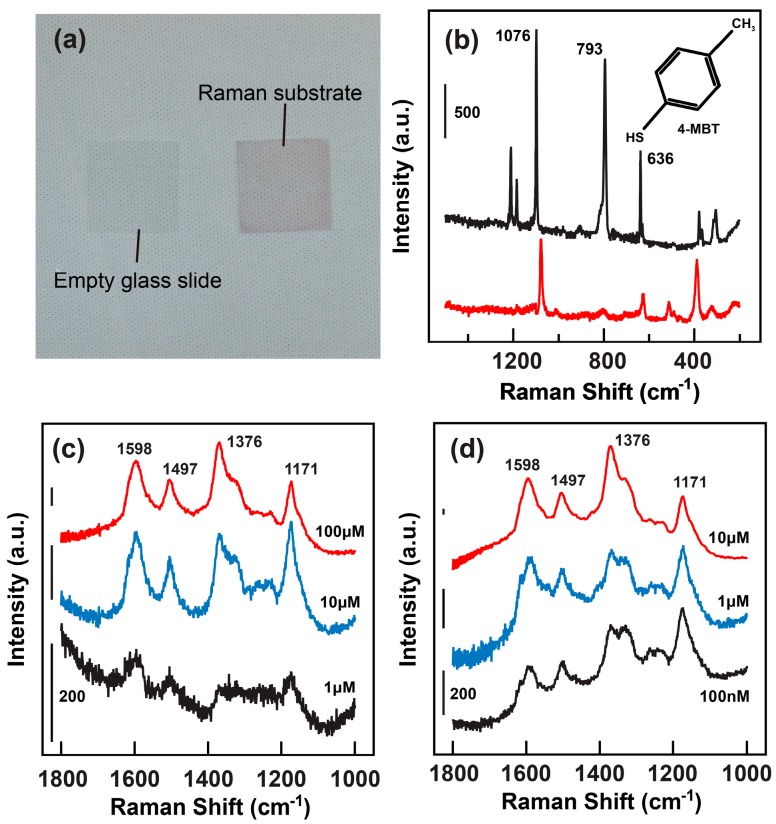
SERS activity of the large-aspect-ratio gold nanorods. (**a**) Digitial photograph of the SERS substrate fabricated with glass slide. (**b**) Raman spectra of the 4-MBT on the SERS substrate (red) and in powder form (black). The molecular formula of the 4-MBT is presented in the inset. The spectrum of the molecular powder was collected using a laser power of 118.00 mW, while that from the SERS substrate was collected with a power of 1.37 mW. (**c**,**d**) Raman spectra of the PANI-ES on the pristine glass substrate and SERS substrate. The baselines of the Raman spectra have been modified for clear demonstration of the data.

SERS is a very important sensing technique that is strongly related to the plasmon resonances of noble metal nanostructures. To demonstrate the SERS performance of the large-aspect-ratio gold nanorods, the nanorods were first immobilized onto the glass slide to form a SERS substrate using a wet-chemical procedure (see Materials and Methods). The photograph of the SERS substrate is given in Figure 3a, which shows an excellent transparency of the substrate upon deposition of the nanorods. The associated AFM image clearly indicates the uniform distribution of the nanorods onto the glass substrate without aggregation (Supplementary Materials, Figure S3). It should be noted that the deposition time of the nanorods is a crucial element for the preparation of the SERS substrate. If the depositing time is too long, the nanorods deposited onto the glass will aggregate to form clusters, which result in a dark red appearance on the glass substrate and lose their SERS activity. However, if the depositing time is short, the nanorods will distribute sparsely onto the glass surface, which cannot provide adequate surface area as well as signal enhancements for the SERS characterizations. After carefully adjusting the experiment conditions, we found that a 6-h immersion of the thiol-functionalized glass substrate into the nanorod aqueous solution can give optimum distributions of the nanorods onto the glass substrate.

The 4-MBT was selected as probe molecules for evaluating the Raman enhancement factor (EF) of the SERS substrate, which can be adsorbed onto the gold surface to form a well-defined monolayer via its thiol group [12]. One advantage of the 4-MBT molecules as SERS probe molecules is that they exhibit clear molecular fingerprints in 400–2000 cm^–1^ spectral region, which can help for precisely calculating the Raman enhancement factor of a SERS substrate. Figure 3b shows the comparison of the Raman spectra of the 4-MBT molecules in powder form and adsorbed onto the nanorod substrate upon excitation with 1064-nm laser, which is in resonance with the longitudinal mode of the nanorod. The laser powers for collecting these two spectra were 118.00 mW and 1.37 mW, respectively. These two spectra showed comparable intensities despite that they were collected with laser powers differing in ~ 100 times. Moreover, the phenyl ring-breathing mode at 1076 cm^–1^ can be well-resolved on the Raman spectrum collected from the nanorod substrate adsorbed with only a monolayer 4-MBT [12]. Such a result clearly indicates that due to the strong electromagnetic field enhancements induced by the plasmon resonances of the gold nanorods, the Raman scattering from the molecules can be enhanced. One should pay attention to the huge differences between the Raman spectra collected from the 4-MBT powder and SERS substrate. The origins of such differences are not so clear yet. We think that they can be attributed to the modification of molecule structures upon adsorption onto the gold nanorod surface. The binding of the –SH group onto the metal surface may induce charge transfer between the 4-MBT molecules and gold nanorod, which will induce polarized charges at the interface and therefore modify the molecular geometry. Such an effect will lead to a much different Raman spectrum.

According to the SERS spectra of the pristine 4-MBT and those measured from the SERS substrate, the EF can be calculated using the following equation [16]:

(3) $$\;\mathrm{EF}=\frac{I_{\mathrm{SERS}}/N_{\mathrm{SERS}}}{I_{\mathrm{Raman}}/N_{\mathrm{Raman}}}\;$$

where *I*_SERS_ and *I*_Raman_ are the Raman intensities of the 1076 cm^−1^ mode obtained from the SERS substrate and pristine 4-MBT powder. Parameter *N*_SERS_ is the number of the molecules on the SERS substrate while the *N*_Raman_ is the amount of 4-MBT molecules in the focal volume of the laser spot. Because the 4-MBT has formed a monolayer on the surface of the gold nanorods, the *N*_SERS_ can then be determined by the overall surface area of the gold nanorods that are exposed in the laser spot, which can be estimated according to the AFM image (Supplementary Materials, Figure S3). The diameter of the incident laser spot is around 1 μm. An individual nanorod is modeled as a block with longitudinal and transverse lengths of 131 nm and 18 nm respectively, and only five facets of nanorod are considered because the rest facet is attached to the glass substrate without adsorption of the molecules. The *N*_Raman_ is equal to *V*_L_/*V*_M_, where *V*_L_ (=1.48 pL) represents the focal volume of the Raman system, and *V*_M_ represents the volume of a single 4-MBT molecule, which equals to the footprint (0.19 nm^2^) multiplied by the thickness (0.5 nm) of the molecule [16,17]. With these parameters, the EF of the large-aspect-ratio nanorods is calculated to be 9.47 × 10^8^. Such an EF is not as high as expected, which is consistent with previous studies on how sizes and shapes of noble nanoparticle affect their SERS sensing capability [18,19].

Usually the Raman intensity decreases with four powers of the excitation wavelength [2]. Therefore, to further demonstrate the SERS activity of the gold nanorods, probe molecules with strong optical absorption in the near-infrared region are preferred. In this consideration, the PANI-ES was chosen as probe molecules and drop-casted onto the pristine glass and SERS substrates. Upon the 1064-nm excitation, the PANI-ES adsorbed onto the pristine substrate exhibit clear Raman bands in the 1000~1800 cm^–1^ region (Figure 3c) [11], which decrease as the molecular concentration is reduced. The Raman signal can hardly be observed when the molecular concentration was below 10 μM. However, for the molecules adsorbed onto the SERS substrate, the characteristic Raman bands can even persist when the molecular concentrations are down to 100 nM (Figure 3d).

**Figure 4:**
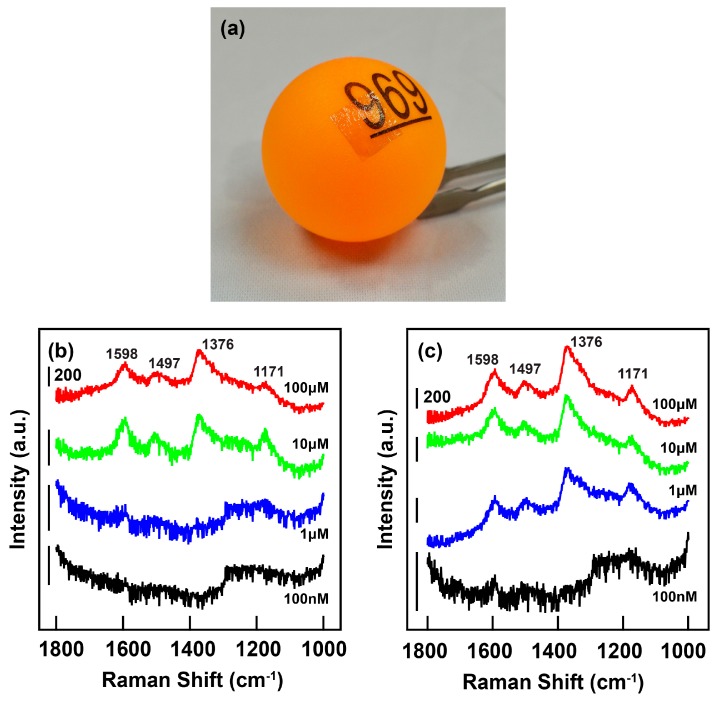
Flexible SERS substrate. (**a**) Digital photograph of the flexbile SERS substrate stuck onto the surface of a table tennis ball; (**b**) Raman spectra of the PANI-ES molecules of different concentrations collected from the empty PS substrate; (**c**) Raman spectra of the PANI-ES molecules of different concentrations collected from the flexible SERS substrate. The excitation wavelength for (**b**,**c**) is 1064 nm. The baselines of the Raman spectra have been modified for better demonstration.

The above results clearly indicate the potential of the gold nanorods with large aspect ratios as SERS substrate. On the other hand, in recent years transparent and flexible SERS substrate that are convenient to carry and can conform to surfaces with random curvature have attracted much attention. They can greatly benefit the in situ detection of toxicants from curved food surfaces [13,20]. We also explored the applications of the large-aspect-ratio gold nanorods in fabrication of the flexible SERS substrate. To that end, the gold nanorods were first deposited onto a glass substrate to form a monolayer with uniform nanorod distribution (Supplementary Materials, Figure S4a). Due to the change of the RI, the longitudinal plasmon resonance of the nanorods shifts from 1176 nm to 1024 nm when they are supported onto the glass substrate (Supplementary Materials, Figure S4a,b). Subsequently a PS toluene solution was spin-coated onto the glass substrate to form a PS thin film. Thereafter, the PS film adhered with the gold nanorods was peeled off from the glass by heating up the substrate (Supplementary Materials, Figure S4b,c), whereby the flexible SERS substrate can be obtained. Due to the increase of the RI by the PS film (1.57), the flexible SERS substrate exhibits a longitudinal plasmon resonance at 1115 nm (Supplementary Materials, Figure S5). As shown in Figure 4a, the flexible SERS substrate can be easily attached onto a table tennis ball with a convex surface. The film exhibits excellent optical transparency as well. The SERS activity of the flexible substrate was characterized by measuring the Raman spectra of the PANI-ES molecules adsorbed on it. As shown in Figure 4b,c, the Raman spectra obtained from the PS film integrated with the gold nanorods are stronger than those from the blank PS film. The Raman fingerprints can be observed when the molecular concentration is as low as 100 nM.

## 4. Conclusions

In summary, we have demonstrated the plasmonic sensing characteristics of the gold nanorods with large aspect ratios. It was shown that nanorods of an aspect ratio of 7.9 exhibited longitudinal plasmon resonance at 1060 nm. Such a large plasmon wavelength gives rise to a RI sensitivity up to 473 nm/RIU, with an FOM of 1.8. The gold nanorods were then employed to fabricate SERS substrate, which showed excellent activity under excitation of 1064 nm. The EF was calculated to be 9.47 × 10^8^. Additionally, a transparent and flexible SERS substrate operating in the near-infrared region was fabricated by integrating the gold nanorods with the PS polymer. We believe that our results pave the way for future high-performance plasmonic sensing techniques that are based on the gold nanorods.

## Supplementary Materials

The following are available online at http://www.mdpi.com/1424-8220/18/10/3458/s1, Figure S1: Schematic showing the procedures of growing the gold nanorods with large aspect ratios; Figure S2: Dependence of the refractive index on the mass ratio of glycerol in water–glycerol mixture; Figure S3: AFM image of the SERS substrate; Figure S4: Schematics showing the fabrication of the flexible SERS substrate; Figure S5: Extinction spectra of aqueous gold nanorods sample, gold nanorods deposited onto glass substrate, and the flexible SERS substrate.
