# Supplementary material for: Plasmonic Sensing Characteristics of Gold Nanorods with Large Aspect Ratios
Source: Sensors (Basel). 2018 Oct 15;18(10):3458. doi: 10.3390/s18103458 (PMC6210301; doi:10.3390/s18103458)
Supplement: Supplementary file 1 [file sensors-18-03458-s001.pdf]

Plasmonic Sensing Characteristics of Gold Nanorods with Large Aspect Ratios

Chao Zhuang, Yifan Xu, Ningsheng Xu, Jinxiu Wen, Huanjun Chen * and Shaozhi Deng *

State Key Laboratory of Optoelectronic Materials and Technologies, Guangdong Province Key Laboratory of Display Material and Technology, Sun Yat-sen University, Guangzhou 510275, China; zhuangch3@mail2.sysu.edu.cn (C.Z.); xuyf35@mail2.sysu.edu.cn (Y.X.); stsxns@mail.sysu.edu.cn (N.X.); jinxiuwen@foxmail.com (J.W.)

* Correspondence: chenhj8@mail.sysu.edu.cn (H.C.); stsdsz@mail.sysu.edu.cn (S.D.); Tel.: +86-20-8411-0916

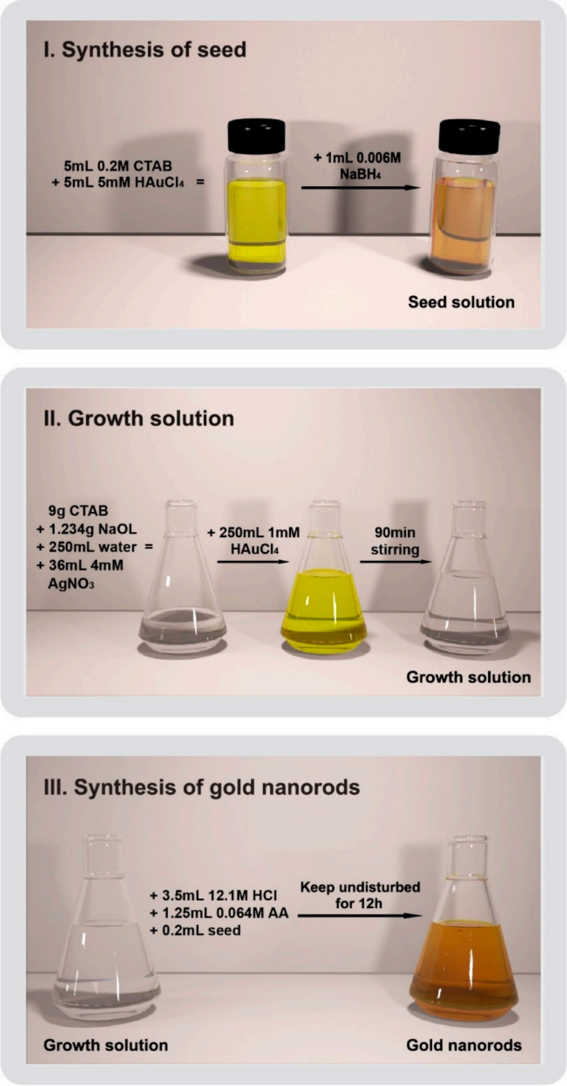

Figure S1. Schematic showing the procedures of growing the gold nanorods with large aspect ratio.

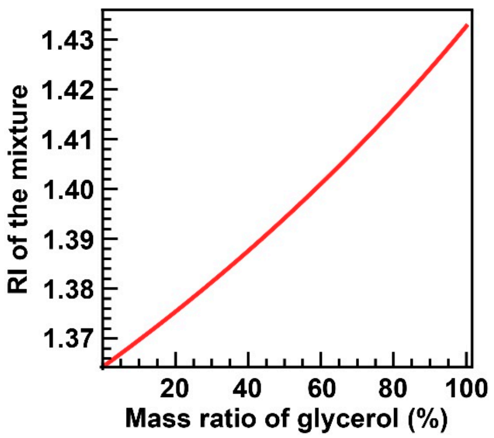

Figure S2. Dependence of the refractive index on the mass ratio of glycerol in water–glycerol mixture.

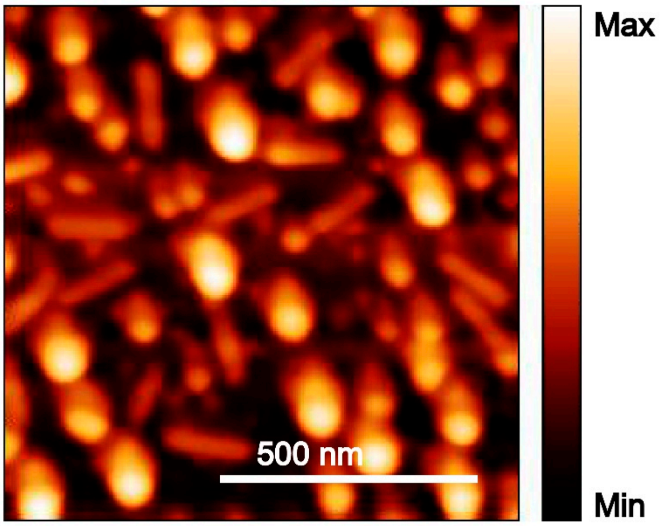

Figure S3. AFM image of the SERS substrate. The SERS substrate sample was used without any further functionalization.

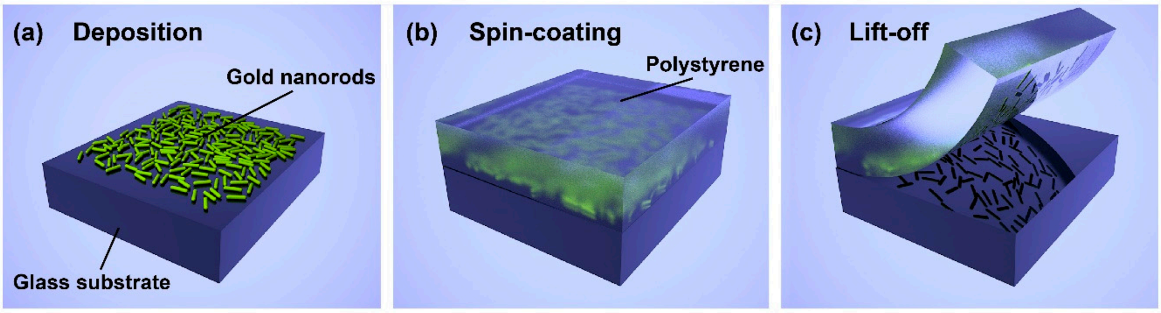

Figure S4. Schematics showing the fabrication of the flexible SERS substrate.

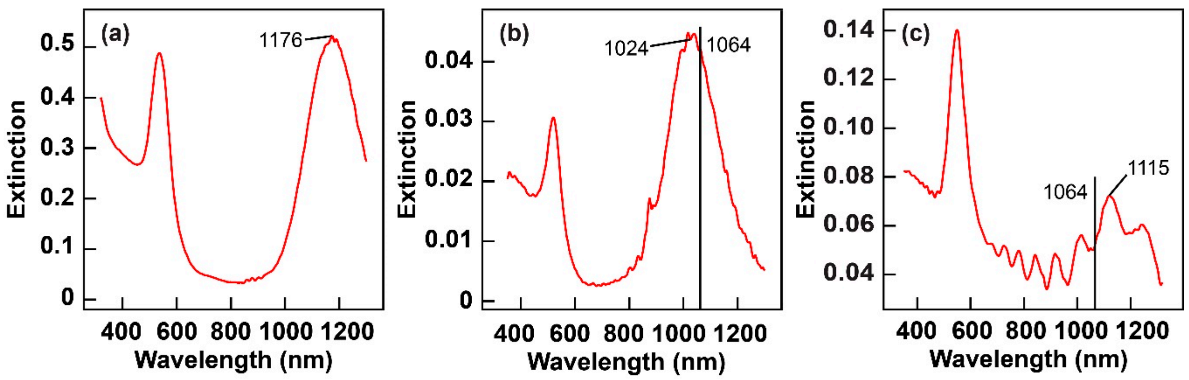

Figure S5. Extinction spectra of (**a**) aqueous gold nanorod sample; (**b**) gold nanorods deposited onto glass substrate; (**c**) flexible SERS substrate.
